# Supplementary material for: Systematic analysis of immune cell motility leveraging the open intravital microscopy database Immunemap
Source: EMBO J. 2025 Nov 20;45(1):334–48. doi: 10.1038/s44318-025-00629-4 (PMC12759063; doi:10.1038/s44318-025-00629-4)
Supplement: Supplementary file 1 — Appendix [file 44318_2025_629_MOESM1_ESM.pdf]

Appendix for:

Systematic analysis of immune cell motility leveraging the open intravital microscopy database Immunemap

Table of contents

Appendix Figure S1. Workflow for generating cell tracks in Immunemap. 2

Appendix Figure S2. Impact of dimensionality reduction on key motility metrics. 3

Appendix Figure S3. Immunemap Architecture. 4

Appendix Figure S4. Tailored motility analysis through the interoperability between immunemap and CelltrackR. 5

Appendix Figure S5. Example applications of computer vision methods to Immunemap. 6

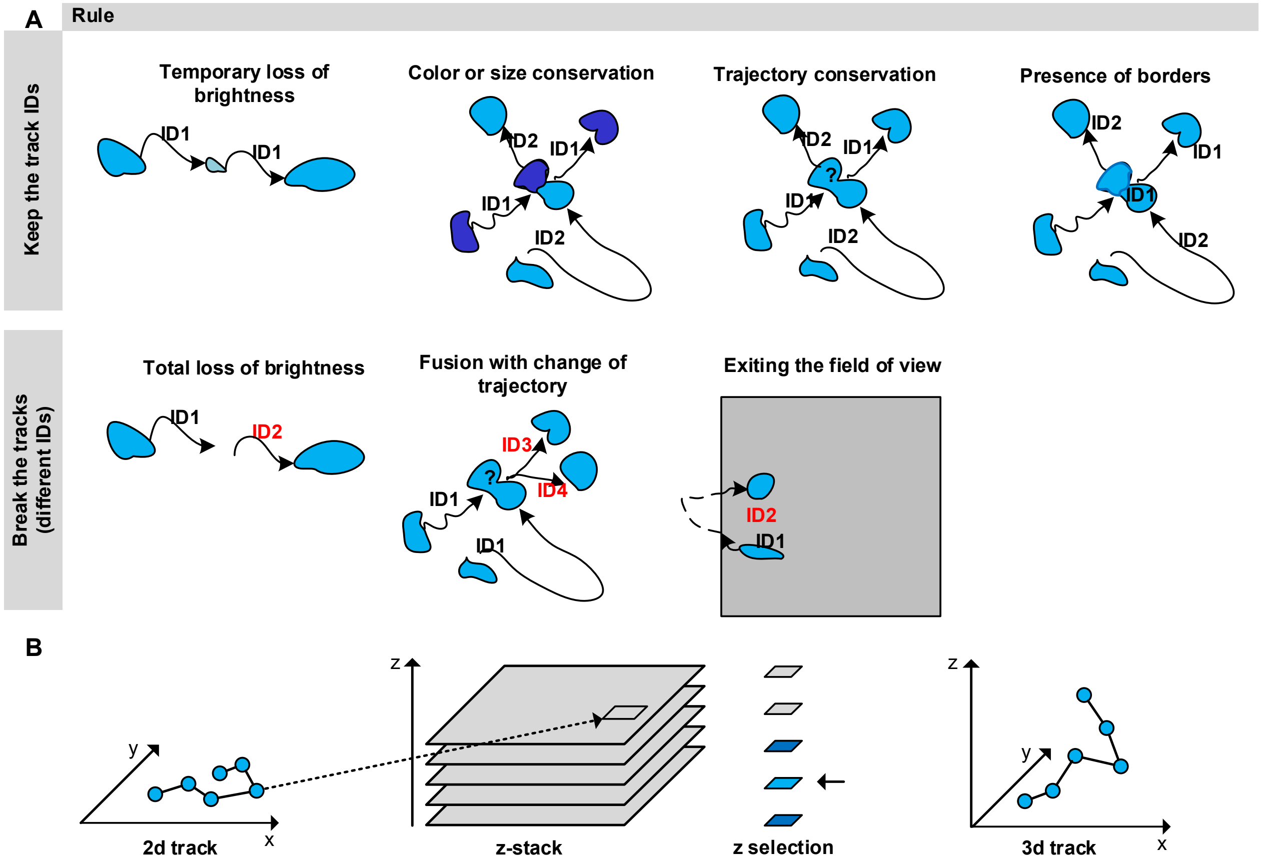

Appendix Figure S1. Workflow for generating cell tracks in Immunemap. Outline of the standardized methodology used to produce accurate and reproducible single-cell tracks for inclusion in the Immunemap database. **A)** Manual 2D and tracking rules for resolving ambiguous cases. Cell tracking was performed manually in 2D by expert operators following consistent guidelines. Tracking was maintained when a cell transiently lost brightness but remained partially visible, or when cells merged yet could still be individually resolved based on color continuity, trajectory coherence, or distinguishable borders. Conversely, tracks were interrupted when cells completely disappeared, merged indistinguishably, or exited the field-of-view. **B)** Automated projection of 2D tracks into 3D space. To reconstruct full 3D trajectories from manually annotated 2D tracks, the corresponding z-plane with maximum fluorescence intensity was automatically identified at each time point—enabling reliable estimation of the z-position throughout the cell's path.

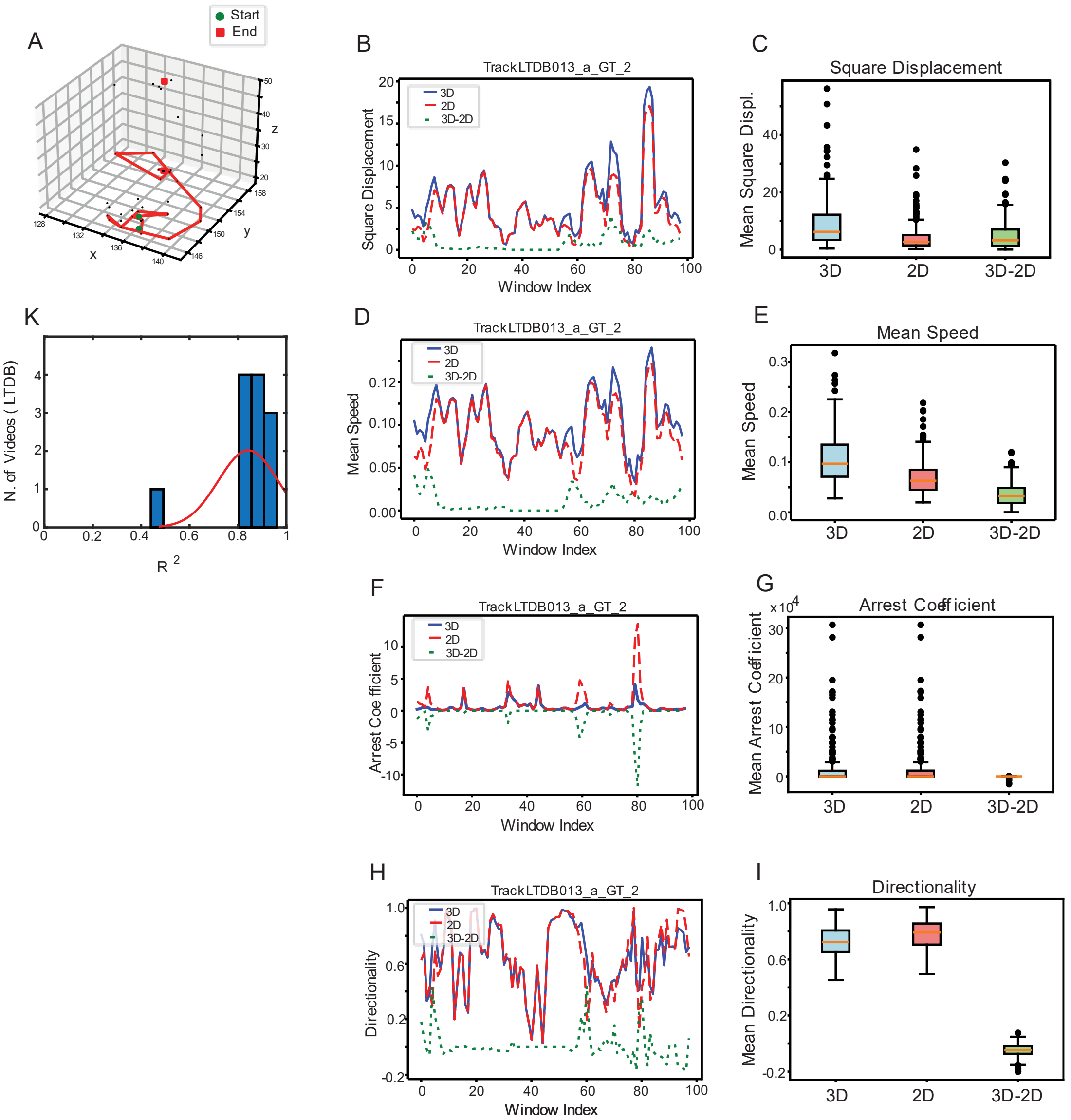

Appendix Figure S2. Impact of dimensionality reduction on key motility metrics. **A)** Graphic representation of a representative 3D immune cell trajectory (blue) and its 2D-projected counterpart (red), generated by collapsing the z-axis. **B-I)** Quantitative comparison of motility features computed from the original 3D track versus its 2D projection (track: LTDB013_a_GT_2, from the LTDB dataset (Pizzagalli *et al*, 2018)). Metrics shown include square displacement **(B, C)**, mean speed **(D, E)**, arrest coefficient **(F, G)**, and directionality **(H, I)**. For each feature, values calculated in 2D (excluding z-axis) are compared with those from the full 3D track (including z-axis). The panels labeled "3D–2D" represent the difference between 3D and 2D values, calculated as: 3D metric – 2D metric, such that negative values indicate overestimation of the metric in 2D relative to 3D. Box plots show the median (red central line), interquartile range (box bounds), minima and maxima (whiskers), and outliers (black dots). n = 313 tracks in each group. **K)** Distribution of the R2 coefficient among 3D videos, showing an average of 0.84.

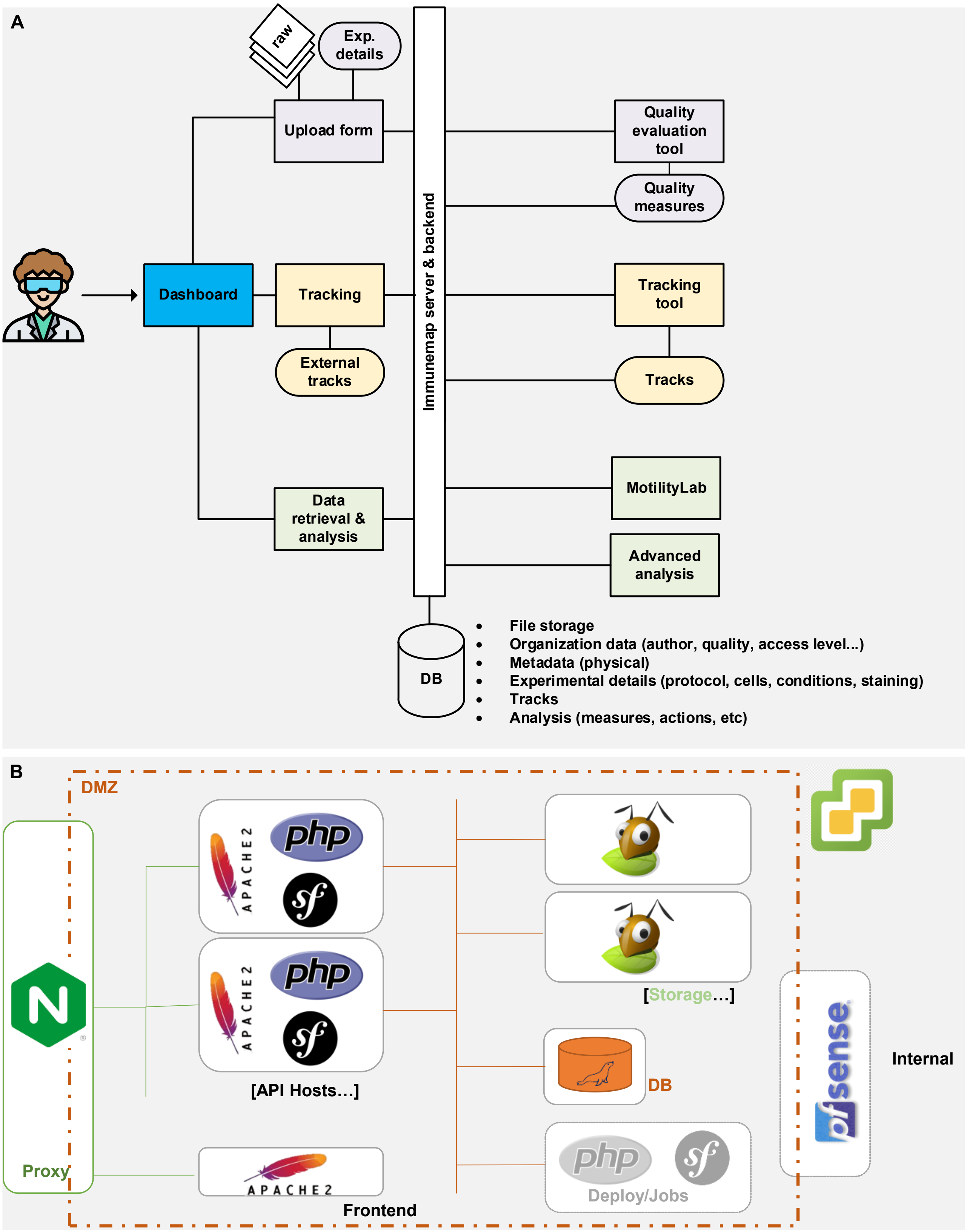

Appendix Figure S3. Immunemap Architecture. **A**) Functionalities provided by the platform. **B**) Internal software components

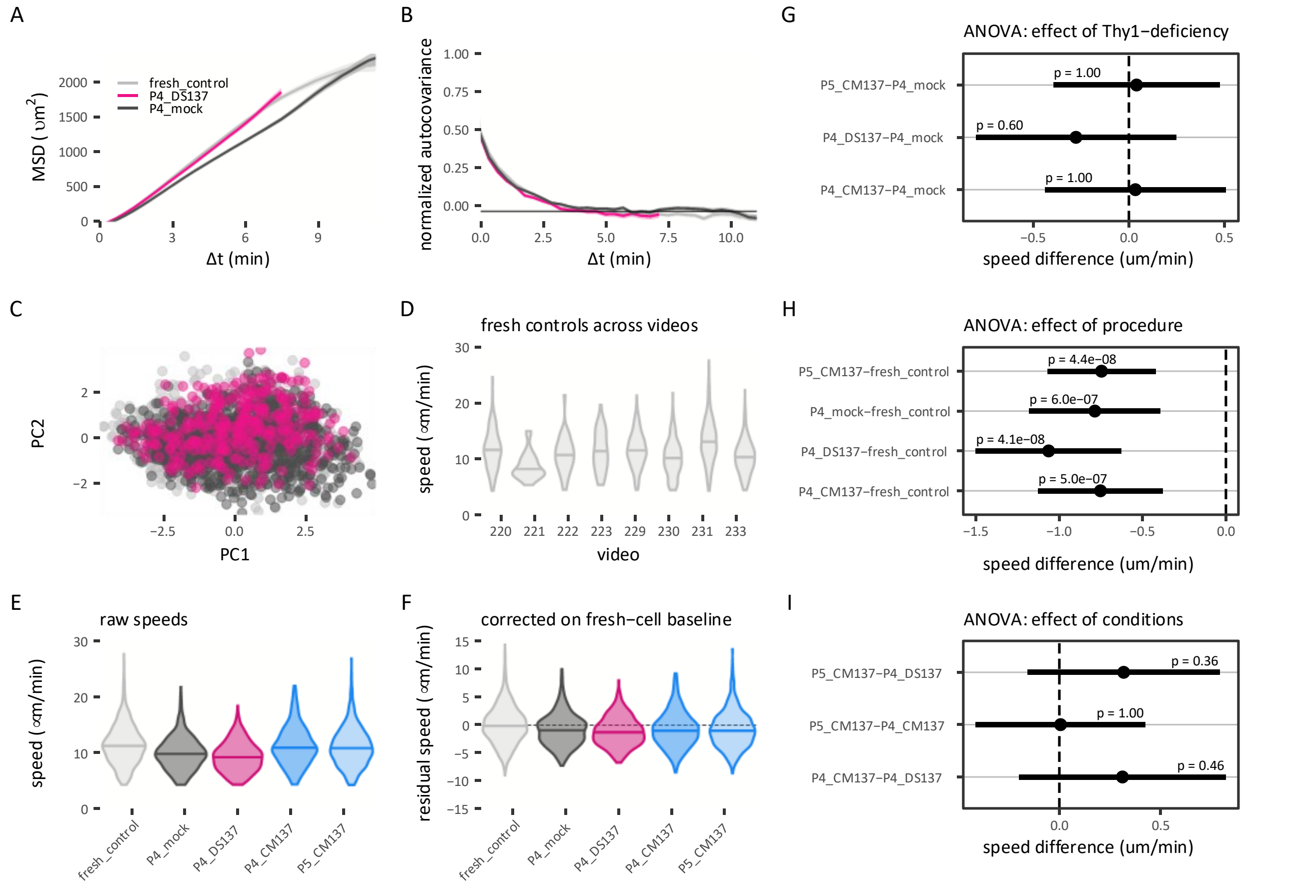

Appendix Figure S4. Tailored motility analysis through the interoperability between immunemap and CelltrackR. Tracks from experiment 48 on immunemap were loaded in CelltrackR. The experiment contains 8 videos of CD8+ naïve T cells in the popliteal lymph node (for details, see immunemap exp 48 at https://app.immunemap.org/experiment-publicview?id=48). Each video contains freshly isolated, reference cells ("fresh control") along with 2 populations that were mock-nucleofected with gRNA against surface antigen Thy1 under several conditions ("P4_mock", "P4_DS137", "P4_CM137", "P5_CM137"). **A-B)** Example mean squared displacement (MSD, **A**) and autocovariance **(B)** curves for the 3 videos that contained fresh control cells, mock-nucleofected cells, and cells nucleofected with pulse DS137, suggesting similar motility with a persistence time of about 5 min. Accordingly, populations did not separate in **C)** a principal component analysis of 5 motility metrics (speed, straightness, sphericity, outreach ratio, and turning angle) computed on tracklets of 20 steps (about 7 min). However, a common problem in track datasets is high inter-video variability as shown by **D)** the speeds (distribution and median) of the freshly isolated control population, which should be (but is not) the same between. Thus, **E)** a comparison of raw track speeds (distribution and median) across all videos is misleading and should **F)** first be corrected by subtracting the mean "baseline speed" of fresh control cells from the same video. After correction, **G-I)** an ANOVA followed by a Tukey test was used to estimate the effects of interest (dots) along with adjusted p-values and a 95% confidence interval (segments). Here, **G)** shows no evidence for an effect of Thy1-deficiency by contrasting different nucleofection conditions against a mock-nucleofected control, **H)** shows that mock-nucleofected cells are about 1 µm/min slower compared to freshly isolated ones, and **I)** shows no evidence for a difference between nucleofection conditions. See also Supplemental file tutorial_celltrackr_subtypesT.html in (Dataset EV1) for an in-depth tutorial reproducing this figure. (n = 309, 27, 41, 55, 386, 194, 345, 455 tracks for each video in panel D, n = 1812, 539, 416, 616, 923 tracks for each group in panels E-I).

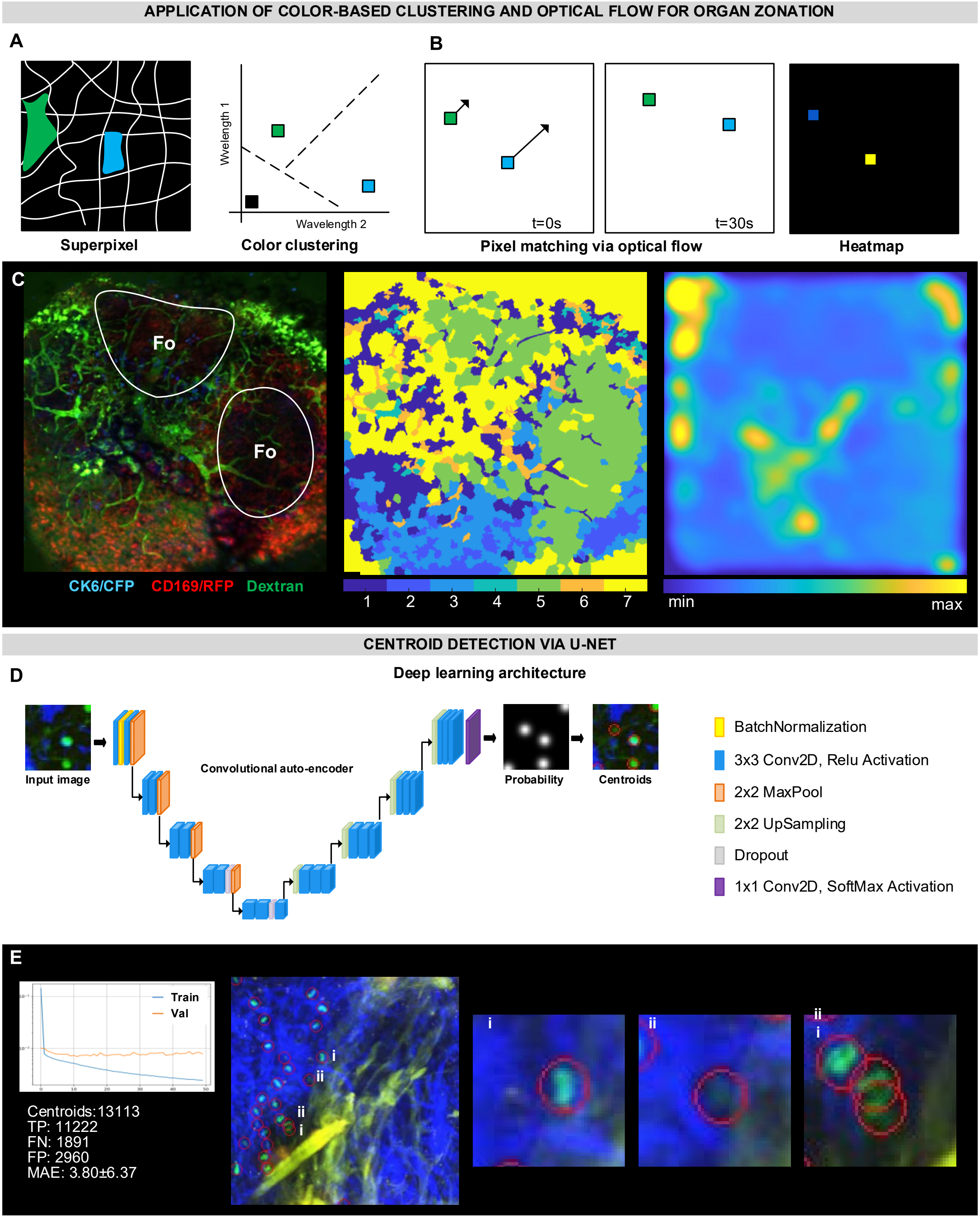

Appendix Figure S5. Example applications of computer vision methods to Immunemap. A) Decomposition of images in superpixel and clustering based on color features. **B)** Matching of pixels at adjacent time points via Optical Flow, creating a heatmap of motility intensity. **C)** Results of superpixel clustering (left) and optical flow (right) applied to a IVM time-lapse capturing CK6/CFP labeled neutrophils in the popliteal lymph node (video id 72, https://app.immunemap.org/acquisition-public-view?id=47&videoID=72). **D)** Autoencoder deep learning architecture for centroid detection in IVM data. E. Performances of the U-NET architecture (left) and selected IVM micrographs reporting the detected centroids (red circles) in case of clearly visible cells (i), cells with low contrast (ii), and cells in contact (iii).
