# Supplementary material for: Systematic analysis of immune cell motility leveraging the open intravital microscopy database Immunemap
Source: EMBO J. 2025 Nov 20;45(1):334–48. doi: 10.1038/s44318-025-00629-4 (PMC12759063; doi:10.1038/s44318-025-00629-4)
Supplement: Supplementary file 3 — Dataset EV1 [file 44318_2025_629_MOESM3_ESM.zip › WEB_PLATFORM/API Documentation.docx]

# **API Documentation**

These are the APIs for<https://api.immunemap.org/>

[GET​/acquisition​/{id}](#_k2mvsm5rc4c9)

[function to get an Acquisition by Acquisition ID](#_k2mvsm5rc4c9)

[GET​/acquisition​/video​/{video}](#_wi9w1ah9vdww)

[function to get an Acquisition by video ID](#_wi9w1ah9vdww)

[GET​/video​/{video}](#_vq8nhc4860kg)

[function to get a video by Video ID](#_vq8nhc4860kg)

[GET​/video​/acquisition​/{acquisition}](#_az0o3bb3phzk)

[function to get a video by Acquisition ID](#_az0o3bb3phzk)

[GET​/video​/{video}​/tracks](#_n2nw4rtzjc7s)

[function to get a video tracks by Video ID](#_n2nw4rtzjc7s)

[GET​/video​/](#_7xfu3ltq8l2z)[​{video}](#_n2nw4rtzjc7s)/[preview](#_7xfu3ltq8l2z)

[Get the video preview file](#_7xfu3ltq8l2z)

# **Acquisition API Documentation**

## **GET /acquisition/{id}**

This endpoint allows you to retrieve information about a specific Acquisition entity by its unique identifier.

### **Resource URL**

<https://api.immunemap.org/acquisition/10>

### **Parameters**

- {id} (required, integer) - The unique identifier of the Acquisition entity to retrieve. The {id} must be a positive integer.

### **Response**

- HTTP Status Code: 200 OK
- Content Type: application/json

#### **Example Response:**

json

{

"id":10

"experimental_group":8

"name":"10 hours post vaccination"

"sequence":1

"area":"Lymph node"

"magnification":25

"notes":"intravital imaging using two-photon microscopy, of NK cells (green) and macrophages (red) in the lymph node, into a Ncr1-GFP animal, after UV-PR8 virus injection (white). "cell_stainings":

"video":17

"video_details":

"DB_id":17

"id":"37AXXPYHHN80XBCD4D57ENTKFA"

"uid":"37AXXPYHHN80XBCD4D57ENTKFA"

"owner":"elisa.palladino@irb.usi.ch"

"author":"elisa.palladino@irb.usi.ch"

"location":"\/srv\/immunemap\/backend\/public\/uploads\/c5637a29-40ed-4d88-89d5-405c5b63a474.data"

"size":

"width":555

"height":555

"slices":16

"frames":30

"spacing":[0.8,0.8,3]

"fps":0.01702620332692

"number_of_channels":4

"channel_configuration":

"deleted":false

"created":

"date":"2022-03-10 14:38:37.000000","timezone_type":3,"timezone":"UTC"

"recorded":

"date":"2014-09-30 16:56:42.000000","timezone_type":3,"timezone":"UTC"

"notes":

"time_steps":

"annotations":[]

"uploaded_from":"10.25.11.22"

"uploaded_on":{"date":"2022-03-10 14:38:37.000000","timezone_type":3,"timezone":"UTC"}

"size":854447056,

"name":"15-27-16.ims"

"upload_status":4

"tags":

{"value":"collagen"}

{"value":"follicular-dendritic-cells"}

{"value":"influenza"}

{"value":"influenza-a"}

{"value":"macrophages"}

{"value":"natural-killer-cells"}

{"value":"vaccination"}]

"links":

"mouseId":"0"

"official_mouse_name":

"gender":"male"

}

### **Authorization**

This endpoint requires the user to have the VIEW permission on the specified Acquisition entity. Users without this permission will receive a 403 Forbidden response.

### **Request Example**

You can make a GET request to this endpoint to retrieve information about a specific Acquisition entity. Replace {id} with the actual identifier of the Acquisition you want to retrieve.

http

GET /acquisition/10

###

### **Error Responses**

- HTTP Status Code: 404 Not Found
  If the specified Acquisition entity with the given ID does not exist, the API will return a 404 Not Found response.
- HTTP Status Code: 403 Forbidden
  If the user does not have the VIEW permission on the specified Acquisition entity, the API will return a 403 Forbidden response.

# **Acquisition by Video ID API Documentation**

## **GET /acquisition/video/{videoId}**

This endpoint allows you to retrieve Acquisitions associated with a specific Video by its unique identifier.

### **Resource URL**

<https://api.immunemap.org/acquisition/video/17>

### **Parameters**

- {videoId} (required, integer) - The unique identifier of the Video entity to retrieve associated Acquisitions. The {videoId} must be a positive integer.

### **Response**

- HTTP Status Code: 200 OK
- Content Type: application/json

#### **Example Response:**

json

{

"id":10

"experimental_group":8

"name":"10 hours post vaccination"

"sequence":1

"area":"Lymph node"

"magnification":25

"notes":"intravital imaging using two-photon microscopy, of NK cells (green) and macrophages (red) in the lymph node, into a Ncr1-GFP animal, after UV-PR8 virus injection (white). "cell_stainings":

"video":17

"video_details":

"DB_id":17

"id":"37AXXPYHHN80XBCD4D57ENTKFA"

"uid":"37AXXPYHHN80XBCD4D57ENTKFA"

"owner":"elisa.palladino@irb.usi.ch"

"author":"elisa.palladino@irb.usi.ch"

"location":"\/srv\/immunemap\/backend\/public\/uploads\/c5637a29-40ed-4d88-89d5-405c5b63a474.data"

"size":

"width":555

"height":555

"slices":16

"frames":30

"spacing":[0.8,0.8,3]

"fps":0.01702620332692

"number_of_channels":4

"channel_configuration":

"deleted":false

"created":

"date":"2022-03-10 14:38:37.000000","timezone_type":3,"timezone":"UTC"

"recorded":

"date":"2014-09-30 16:56:42.000000","timezone_type":3,"timezone":"UTC"

"notes":

"time_steps":

"annotations":[]

"uploaded_from":"10.25.11.22"

"uploaded_on":{"date":"2022-03-10 14:38:37.000000","timezone_type":3,"timezone":"UTC"}

"size":854447056,

"name":"15-27-16.ims"

"upload_status":4

"tags":

{"value":"collagen"}

{"value":"follicular-dendritic-cells"}

{"value":"influenza"}

{"value":"influenza-a"}

{"value":"macrophages"}

{"value":"natural-killer-cells"}

{"value":"vaccination"}]

"links":

"mouseId":"0"

"official_mouse_name":

"gender":"male"

}

### **Authorization**

This endpoint requires the user to have the VIEW permission on the specified Video entity. Users without this permission will receive a 403 Forbidden response.

### **Request Example**

You can make a GET request to this endpoint to retrieve Acquisitions associated with a specific Video. Replace {videoId} with the actual identifier of the Video you want to retrieve acquisitions for.

http

GET /acquisition/video/57

###

### **Error Responses**

- HTTP Status Code: 404 Not Found
  If the specified Video entity with the given ID does not exist, the API will return a 404 Not Found response.
- HTTP Status Code: 403 Forbidden
  If the user does not have the VIEW permission on the specified Video entity, the API will return a 403 Forbidden response.

# **Video by Video ID API Documentation**

## **GET /video/{videoId}**

This endpoint allows you to retrieve information about a specific Video entity by its unique identifier.

### **Resource URL**

bash

<https://api.immunemap.org/video/17>

### **Parameters**

- {videoId} (required, integer) - The unique identifier of the Video entity to retrieve. The {videoId} must be a positive integer.

### **Response**

- HTTP Status Code: 200 OK
- Content Type: application/json

#### **Example Response:**

json

{

"DB_id":17

"id":"37AXXPYHHN80XBCD4D57ENTKFA"

"uid":"37AXXPYHHN80XBCD4D57ENTKFA"

"owner":"elisa.palladino@irb.usi.ch"

"author":"[elisa.palladino@irb.usi.ch](mailto:elisa.palladino@irb.usi.ch)”

"location":"\/srv\/immunemap\/backend\/public\/uploads\/c5637a29-40ed-4d88-89d5-405c5b63a474.data"

"video_details":

"size":

"width":555

"height":555

"slices":16

"frames":30

"spacing":[0.8,0.8,3]

"fps":0.01702620332692

"number_of_channels":4

"channel_configuration":

"deleted":false

"created":

"date":"2022-03-10 14:38:37.000000","timezone_type":3,"timezone":"UTC"

"recorded":

"date":"2014-09-30 16:56:42.000000","timezone_type":3,"timezone":"UTC"

"notes":

"time_steps":

"annotations":[]

"uploaded_from":"10.25.11.22"

"uploaded_on":{"date":"2022-03-10 14:38:37.000000","timezone_type":3,"timezone":"UTC"}

"size":854447056,

"name":"15-27-16.ims"

"upload_status":4

}

### **Authorization**

This endpoint requires the user to have the VIEW permission on the specified Video entity. Users without this permission will receive a 403 Forbidden response.

### **Request Example**

You can make a GET request to this endpoint to retrieve information about a specific Video entity. Replace {videoId} with the actual identifier of the Video you want to retrieve.

http

GET /video/17

### **Error Responses**

- HTTP Status Code: 404 Not Found
  If the specified Video entity with the given ID does not exist, the API will return a 404 Not Found response.
- HTTP Status Code: 403 Forbidden
  If the user does not have the VIEW permission on the specified Video entity, the API will return a 403 Forbidden response.

# **Video by Acquisition ID API Documentation**

## **GET video/acquisition/{acquisitionId}**

This endpoint allows you to retrieve the Video associated with a specific Acquisition entity by its unique identifier.

### **Resource URL**

<https://api.immunemap.org/video/acquisition/10>

### **Parameters**

- {acquisitionId} (required, integer) - The unique identifier of the Acquisition entity for which you want to retrieve the associated Video. The {acquisitionId} must be a positive integer.

### **Response**

- HTTP Status Code: 200 OK
- Content Type: application/json

#### **Example Response:**

json

{

"DB_id":17

"id":"37AXXPYHHN80XBCD4D57ENTKFA"

"uid":"37AXXPYHHN80XBCD4D57ENTKFA"

"owner":"elisa.palladino@irb.usi.ch"

"author":"[elisa.palladino@irb.usi.ch](mailto:elisa.palladino@irb.usi.ch)”

"location":"\/srv\/immunemap\/backend\/public\/uploads\/c5637a29-40ed-4d88-89d5-405c5b63a474.data"

"video_details":

"size":

"width":555

"height":555

"slices":16

"frames":30

"spacing":[0.8,0.8,3]

"fps":0.01702620332692

"number_of_channels":4

"channel_configuration":

"deleted":false

"created":

"date":"2022-03-10 14:38:37.000000","timezone_type":3,"timezone":"UTC"

"recorded":

"date":"2014-09-30 16:56:42.000000","timezone_type":3,"timezone":"UTC"

"notes":

"time_steps":

"annotations":[]

"uploaded_from":"10.25.11.22"

"uploaded_on":{"date":"2022-03-10 14:38:37.000000","timezone_type":3,"timezone":"UTC"}

"size":854447056,

"name":"15-27-16.ims"

"upload_status":4

}

### **Authorization**

This endpoint requires the user to have the VIEW permission on the associated Video entity. Users without this permission will receive a 403 Forbidden response.

### **Request Example**

You can make a GET request to this endpoint to retrieve the Video associated with a specific Acquisition entity. Replace {acquisitionId} with the actual identifier of the Acquisition entity for which you want to retrieve the associated Video.

http

GET video/acquisition/10

### **Error Responses**

- HTTP Status Code: 404 Not Found
  If the specified Acquisition entity with the given ID does not exist or does not have an associated Video, the API will return a 404 Not Found response.
- HTTP Status Code: 403 Forbidden
  If the user does not have the VIEW permission on the associated Video entity, the API will return a 403 Forbidden response.

# **Video Tracks by Video ID API Documentation**

## **GET /video/{videoId}/tracks**

This endpoint allows you to retrieve the tracks associated with a specific Video entity by its unique identifier.

### **Resource URL**

<https://api.immunemap.org/video/17/tracks>

### **Parameters**

- {videoId} (required, integer) - The unique identifier of the Video entity for which you want to retrieve the associated tracks. The {videoId} must be a positive integer.

### **Response**

- HTTP Status Code: 200 OK
- Content Type: application/json

#### **Example Response:**

json

{

"id":44639

"label":"15-27-16_1ime0000_ct1.csv_0"

"color":"#FFFF00"

"author":6

"video":17

"date":{"date":"2022-06-29 00:00:00.000000","timezone_type":3,"timezone":"UTC"}

"points":[[0,6.5,459.5,1],[1,3.25,463.25,1],[2,4.25,467.75,1],[3,3.25,478.5,1],[4,3,479.25,1]]

"cellTypeObject":null

"cellTypeName":"NK cells"

}

### **Authorization**

This endpoint requires the user to have the VIEW permission on the specified Video entity. Users without this permission will receive a 403 Forbidden response.

###

### **Request Example**

You can make a GET request to this endpoint to retrieve the tracks associated with a specific Video entity. Replace {videoId} with the actual identifier of the Video for which you want to retrieve tracks.

http

GET /video/17/tracks

### **Error Responses**

- HTTP Status Code: 404 Not Found
  If the specified Video entity with the given ID does not exist, or it does not have associated tracks, the API will return a 404 Not Found response.
- HTTP Status Code: 403 Forbidden
  If the user does not have the VIEW permission on the specified Video entity, the API will return a 403 Forbidden response.

# **Video Preview File API Documentation**

## **GET /video/{videoId}/preview**

This endpoint allows you to retrieve the preview file of a specific Video entity by its unique identifier.

### **Resource URL**

<https://api.immunemap.org/video/17/preview>

### **Parameters**

- {videoId} (required, integer) - The unique identifier of the Video entity for which you want to retrieve the preview file. The {videoId} must be a positive integer.

### **Response**

- HTTP Status Code: 200 OK
- Content Type: video/mp4 (MPEG-4 video file)

### **Authorization**

This endpoint requires the user to have the VIEW permission on the specified Video entity. Users without this permission will receive a 403 Forbidden response.

### **Request Example**

You can make a GET request to this endpoint to retrieve the preview file of a specific Video entity. Replace {videoId} with the actual identifier of the Video for which you want to retrieve the preview file. You can also include the forceCreate query parameter if needed.

http

GET /video/17/preview

###

### **Response**

If the request is successful, you will visualize a video player with the preview.

**Error Responses**

- HTTP Status Code: 404 Not Found
  If the specified Video entity with the given ID does not exist, the API will return a 404 Not Found response.
- HTTP Status Code: 403 Forbidden
  If the user does not have the VIEW permission on the specified Video entity, the API will return a 403 Forbidden response.
- HTTP Status Code: 500 Internal Server Error
  If an error occurs during the retrieval or creation of the preview file, the API will return a 500 Internal Server Error response.
