# Supplementary material for: Systematic analysis of immune cell motility leveraging the open intravital microscopy database Immunemap
Source: EMBO J. 2025 Nov 20;45(1):334–48. doi: 10.1038/s44318-025-00629-4 (PMC12759063; doi:10.1038/s44318-025-00629-4)
Supplement: Supplementary file 3 — Dataset EV1 [file 44318_2025_629_MOESM3_ESM.zip › IMMUNEMAP_FORM/Supplementery_Table_1.pdf]

IMMUNEMAP FORM

*Please fill out this form for each experiment related to the shared videos. Only the fields marked with " * " are mandatory.*

INFORMATION RELATED TO THE EXPERIMENT

You can write the general information related to a specific experiment. In the "Acquisitions" session, you will be able to provide us with the specific information for each acquisition

Name of the experiment*<br><br>*(please write it with the keywords of your experiment. E.g.: IFN-γ production by NK cells)*	
Acquisition date*<br><br>(*When the experiment was performed, mm/dd/yyyy*)	
Organization*<br><br>*(example: IRB, Institute for Research in Biomedicine, Bellinzona, CH)*	
Microscope*<br>(only if not already in the system)	Microscope name*:<br>Number of lasers*:<br>Manufacturer*:<br>Description:
Model*<br><br>(only if not already in the system)<br><br>(*please, specify here the experimental procedure used to prepare the samples. E.g.: Model used for Popliteal lymph node based on Mempel 2004, or any other not standardized procedure used)*	Name*:<br><br>In vivo, in vitro, explant*:<br><br>DOI or link<br>*(related to the experimental procedure used):*<br><br>Description*:
Author (one or more)*<br><br>*(the information of the person who performed the experiment and/or took the acquisitions)*	First name*:<br>Last name*:<br>e-mail*:
Rationale*<br>(please, provide the purpose of this experiment)	
DOI or link:<br><br>*(if it is available, insert here the references related to these videos or the DOI of the publication)*	

IMAGING PROTOCOL

Table:

We aim to include in this table all the objects visible in the experiment acquisitions, so anyone can understand what they are seeing.
There is a default list of imaged entities: immune cell, drug, virus, bacteria, structural tissue, vehicle, tumour.
These entities are subdivided into classes, e.g., different types of immune cells (B cell, eosinophil…), virus (influenza, HIV…), structural tissue (collagen, epithelial, blood vessel, stromal cell), vehicle (nanoparticle, liposome, antibody), tumour (lymphoma, melanoma, breast cancer), etc.
Then, we specify the labelling details: i) labelled molecule/structure, ii) labelling method (reporter gene, dye, antibody, autofluorescence…) and iii) fluorophore. It is possible to include several labelled molecules/structures per imaged entity. For example, for dendritic cells, you could be using CD11C and CD11B, whereas for B cells it could be B220 and CD19.
Additionally, we specify the channel(s) in which each fluorophore is visible, and the laser and filter corresponding to each channel (options pre-defined when creating the microscope).
It is possible to include additional tags (e.g., strain) and provide further details in the description (optional field).

Example:

**Imaged entity**	**Class**	**Molecule/ Structure**	**Labelling Method**	**Fluorophore**	**Ch1**	**Ch2**	**Ch3**	**Other tags**
Immune cells	Neutrophils	CK6	Reporter gene	ECFP			x	CK6-ECFP
		Cytoplasm	Dye	CMTMR	x			celltracker
Virus	HIV	Gag	Reporter gene	GFP		x		

*

**Imaged entity**	**Class**	**Molecule/ Structure**	**Labelling Method**	**Fluorophore**	**Ch1**	**Ch2**	**Ch3**	**Ch4**	**Other tags**

ACQUISITIONS

It is possible to create one or more groups (e.g., control, treatment…) to organize the acquisitions. Each group is defined by its name. Additionally, it is possible to add a description and other tags. We assign each acquisition to its corresponding group.

ACQUISITION ID *(original file name)**	
Group<br>(*optional, e.g.: control, treatment…)*	
Name<br>*(please write it with the keywords of your acquisition. E.g.: acquisition 10 hours after vaccination; acquisition that shows the extravasation of...):*	
Sample ID<br>(*an integer number to identify mice, microwells…*):	
Area of acquisition<br>(*for in vivo/explant, more specific than imaging site. E.g., imaging site could be popliteal lymph node and area of acquisition could be medulla*):	
Gender (*optional; M/F*):	
**Description/Annotations**<br><br>Please provide here a description of the acquisition.<br>*E.g. : intravital imaging of NK cells in healthy mouse liver by intravenous administration of NK1.1 antibody(red) using two-photon microscopy.*<br><br>Here is also possible to add some **notes** related to specific events shown in the acquisition (such as swarming, patrolling etc..)	
